# Treatment of Vertigo: A Randomized, Double-Blind Trial Comparing Efficacy and Safety of *Ginkgo biloba* Extract EGb 761 and Betahistine

**DOI:** 10.1155/2014/682439

**Published:** 2014-06-25

**Authors:** Larysa Sokolova, Robert Hoerr, Tamara Mishchenko

**Affiliations:** ^1^Faculty of Neurology, Bohomolets National Medical University, Shevchenko Avenue, 13, Kiev 01601, Ukraine; ^2^Clinical Research Department, Dr. Willmar Schwabe GmbH & Co. KG, Willmar-Schwabe-Straße 4, 76227 Karlsruhe, Germany; ^3^Institute of Neurology, Psychiatry and Narcology of the NAMS of Ukraine SI, 46, Akademika Pavlova Street, Kharkiv 61068, Ukraine

## Abstract

A multicenter clinical trial was performed to compare the efficacy and safety of *Ginkgo biloba* extract EGb 761 and betahistine at recommended doses in patients with vertigo. One hundred and sixty patients (mean age 58 years) were randomly assigned to double-blind treatment with EGb 761 (240 mg per day) or betahistine (32 mg per day) for 12 weeks. An 11-point numeric analogue scale, the Vertigo Symptom Scale—short form, the Clinical Global Impression Scales and the Sheehan Disability Scale were used as outcome measures. Both treatment groups were comparable at baseline and improved in all outcome measures during the course of treatment. There was no significant intergroup difference with regard to changes in any outcome measure. Numerically, improvements of patients receiving EGb 761 were slightly more pronounced on all scales. Clinical global impression was rated “very much improved” or “much improved” in 79% of patients treated with EGb 761 and in 70% receiving betahistine. With 27 adverse events in 19 patients, EGb 761 showed better tolerability than betahistine with 39 adverse events in 31 patients. In conclusion, the two drugs were similarly effective in the treatment of vertigo, but EGb 761 was better tolerated. This trial is registered with controlled-trials.com ISRCTN02262139.

## 1. Introduction

Dizziness is a symptom reported frequently in primary care, more often by women than men. In a nationally representative sample of 4869 adults in Germany, aged 18 to 79 years, the one-year prevalence of moderate to severe dizziness (including vestibular and nonvestibular vertigo) was 22.9% and the one-year prevalence of moderate to severe vestibular vertigo was 4.9% [1]. The one-year incidence of moderate to severe dizziness was 3.1% and the one-year incidence of moderate to severe vestibular vertigo was 1.4%. The lifetime prevalence of dizziness-related medical consultations amounted to 17.1% [1]. In a cross-national survey of emergency department visits in the United States, 3.3% of cases presented with dizziness. Of these, 32.9% were of otological/vestibular origin and 4% were due to cerebrovascular disease [2]. Central-vestibular vertigo (12.4%), bilateral peripheral vestibulopathy (5.1%), and paroxysmal dysfunction of the vestibular nerve or vestibular organs (3.9%) are among the frequent types of vertigo. In 3.3% of patients, the cause of vertigo remains unclear [3]. Vertigo is frequent in patients with cerebrovascular disease. Compromised blood supply in the vertebrobasilar region has been reported to manifest itself by isolated vertigo in 24% of patients [4] and 17% of patients with cerebral microangiopathy complained of vertigo [5].

In vertigo associated with cerebrovascular disorders, drugs that improve cerebral blood flow are often prescribed. The international survey found betahistine to be the most frequently prescribed drug for the treatment of various kinds of vertigo, including Ménière's disease, benign paroxysmal positional vertigo, other peripheral vertigo, and peripheral vertigo of unknown origin, followed by piracetam and* Ginkgo biloba* extract [6]. Betahistine is a histamine analogue with agonistic activity at the H_1_ and antagonistic activity at the H_3_ histamine receptors. Its efficacy in the treatment of Ménière's disease and other vertiginous syndromes has been demonstrated by randomized, placebo-controlled trials [7, 8].

*Ginkgo biloba* extract EGb 761 enhances cerebral and vestibular blood flow [9, 10] by decreasing blood viscosity [11]. It improves neuronal plasticity [12] as well as mitochondrial function and energy metabolism [13] and protects neurons from oxidative damage [14]. Its efficacy in the treatment of vestibular and nonvestibular vertigo has also been proven by randomized, placebo-controlled trials [15].

The present study was conducted to compare efficacy and safety of EGb 761 to that of the most frequently prescribed antivertigo agent, betahistine, in patients with vertiginous syndromes.

## 2. Patients and Methods

This randomized, placebo-controlled, double-blind, multicenter clinical trial was conducted by outpatient clinics (mostly associated with departments of neurology) at 10 hospitals in Ukraine in accordance with the Declaration of Helsinki, the Guideline for Good Clinical Practice (GCP) of the International Conference on Harmonization (ICH), and applicable local laws. The protocol was approved by the ethics committee of the Ministry of Healthcare of Ukraine and the local ethics committees of the participating sites; it was registered under number ISRCTN02262139 before enrollment of the patients started. Informed consent was obtained from all patients before any trial-related procedures were undertaken.

### 2.1. Patient Selection

Patients of either sex, at least 45 years old, were eligible if they were diagnosed with peripheral vertigo not otherwise specified (H81.3) or vertiginous syndrome not otherwise specified (H81.9) as classified by the International Classification of Diseases, 10th edition (ICD-10) [16], had symptoms of vertigo for at least 3 months, scored at least 3 on a one-to-ten numeric analogue scale (NAS) at screening, and had sufficient Russian or Ukrainian language skills to respond to interview questions and complete questionnaires. A negative pregnancy test and adequate contraception were required from female patients. Patients with specific vertiginous syndromes (e.g., Ménière's disease, Lermoyez syndrome, and benign paroxysmal positional vertigo), vertigo due to specified somatic diseases (except cerebrovascular disease), severe other disorders, contraindications to one of the drugs under study, need for drugs that might interfere with the efficacy assessments, or gastrointestinal disorders with uncertain absorption of the active agents were excluded from the study.

### 2.2. Randomization and Treatment

Randomization stratified by centers was carried out by the sponsor's biometrics department using a validated computer program that matched treatments to drug numbers in a 1 : 1 ratio. Blinding was achieved by a double-dummy technique: that is, all patients received the same number of film-coated tablets (EGb 761 or placebo) and capsules (betahistine or placebo) in a way that each patient received only one active drug. Drug and placebo tablets and drug and placebo capsules, respectively, were indistinguishable in appearance and taste; all packages and labels were identical except for the drug numbers. Each patient was handed the drug package with the lowest drug number still available at the recruiting site. This procedure guaranteed blinding of patients, investigators, and site staff, concealment of allocation, and balance of treatment group sizes.

The treatment period was 12 weeks, during which patients took either 240 mg per day (120 mg b.i.d.)* Ginkgo biloba* extract EGb 761 or 32 mg per day (16 mg b.i.d.) betahistine dihydrochloride. EGb 761 is a dry extract from* Ginkgo biloba* leaves (35–67 : 1), extraction solvent: acetone 60% (w/w) (manufacturer: Dr. Willmar Schwabe GmbH & Co. KG, Karlsruhe, Germany; EGb 761 is a trade mark of Dr. Willmar Schwabe GmbH & Co. KG). The extract is adjusted to 22.0–27.0% ginkgo flavonoids calculated as ginkgo flavone glycosides and 5.0–7.0% terpene lactones consisting of 2.8–3.4% ginkgolides A, B, and C and 2.6–3.2% bilobalide and contains less than 5 ppm ginkgolic acids. The doses of both drugs were chosen in accordance with available evidence of efficacy derived from systematic reviews [7, 8, 15].

### 2.3. Visits and Assessments

To verify the diagnosis for inclusion and the criteria for eligibility, the medical history was recorded and a general physical examination, laboratory tests, and a clinical neurootological examination, including the Romberg test, the Unterberger stepping test, and an evaluation of spontaneous nystagmus with the aid of Frenzel glasses were performed. Assessments of efficacy and safety of the drugs were scheduled 4, 8, and 12 weeks after the baseline visit. Efficacy was evaluated using an eleven-point numeric analogue scale (NAS) with 0 indicating the absence of vertigo and 10 representing extremely severe vertigo, the short form of the Vertigo Symptom Scale (VSS-SF) [17], the Sheehan Disability Scale (SDS) [18], and the Clinical Global Impressions (CGI) Scale [19]. The 15-item VSS-SF is a self-rating scale taking into account frequency and severity of vertigo within the last month. It consists of two subscales to assess two dimensions of vertigo: vertigo-balance (VSS-V) and autonomic-anxiety (VSS-A) symptoms. The maximum score of 60 indicates the most severe symptoms. The SDS is a 3-item self-rating inventory originally designed to assess to what extent psychological symptoms disrupt a patient's work, social life, and family life. It has been used successfully in somatic diseases with emotional distress. Higher scores (maximum: 10) indicate more severe impairment. Of the CGI, items 2 (change) and 3 (therapeutic index) were rated by the investigators following interviews with the patients. To monitor the safety of the treatments, vital signs were examined at all visits and physical examination, 12-lead ECG, and laboratory tests were performed at the screening and final visits. All adverse events experienced by patients during the treatment period and a subsequent two-day washout period were recorded and assessed for seriousness, severity, and causality.

### 2.4. Statistical Analyses

For each of the efficacy variables, the EGb 761 group was compared to the betahistine group with methods of descriptive data analysis. Standard summary statistics (arithmetic mean and standard deviation) were calculated for all quantitative variables. Categorical values are presented in frequency tables including absolute and relative frequencies. Descriptive *P* values were calculated using Wilcoxon tests and Fisher's exact tests for quantitative and categorical parameters, respectively. Efficacy analyses were based on the full analysis data set including all patients who received randomized study treatment at least once and having at least one measurement of any efficacy parameter during the randomized treatment period. Additionally prespecified subgroups (age, gender, clinical neurootological findings, and severity of symptoms) were analyzed. Safety variables were evaluated for the safety population which included all patients randomized to study treatment and who took study medication at least once. Adverse events were summarized by means of appropriate frequency tables based on coded items and taking into account severity and relationship to study drug. Overall incidence rates were compared between treatment groups. Due to the exploratory nature of the study, no formal sample size calculation was performed. The sample size of 2 × 80 = 160 patients was considered to be large enough to allow for a valid comparison of the EGb 761 and the betahistine group with respect to efficacy and safety.

## 3. Results and Discussion

**Figure 1:**
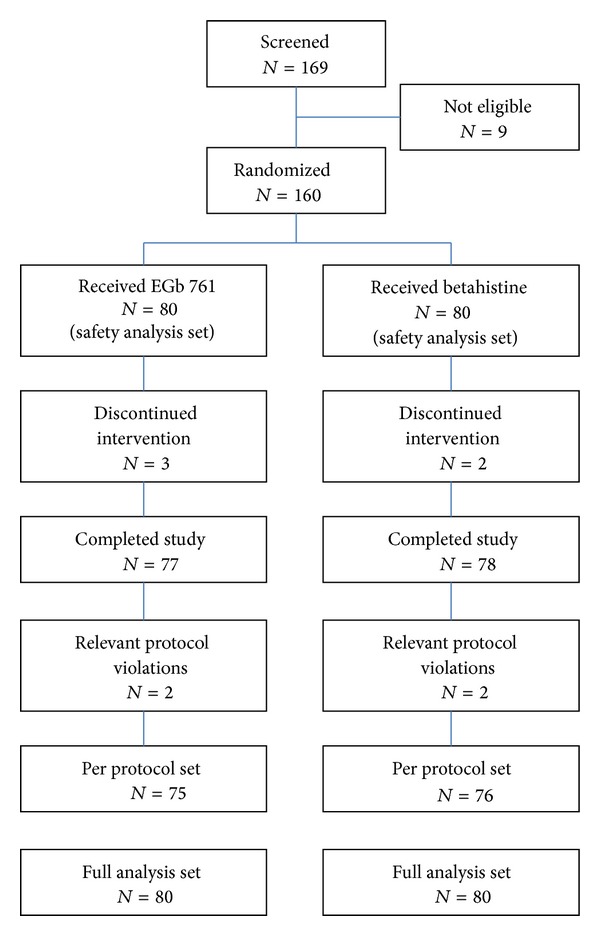
Patient disposition and analysis sets.

Of 169 patients screened, 160 were eligible, received treatment as randomly allocated, and were included in the full analysis set (EGb 761, 80 patients; betahistine, 80 patients). Three patients in the EGb 761 group (unexpected improvement/remission, 1 patient; violation of inclusion/exclusion criteria, 2 patients) and two patients in the betahistine group (withdrawal of informed consent without giving the reason) terminated the study prematurely. Patient disposition and analysis sets are depicted in Figure 1. Demographic data, rating scale scores, and clinical neurootological findings at enrollment are presented in Table 1. There were no conspicuous differences between the treatment groups when treatment was started.

### 3.1. Efficacy

**Figure 2:**
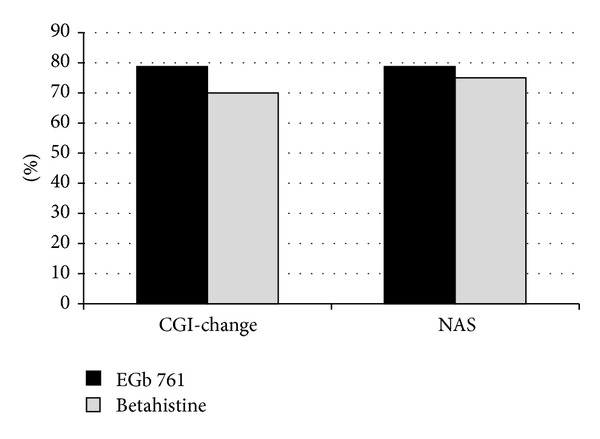
Response rates, defined as clinician's global impression (CGI) rated “much improved” or “very much improved” or patient's rating of NAS improvement at least 50%.

**Figure 3:**
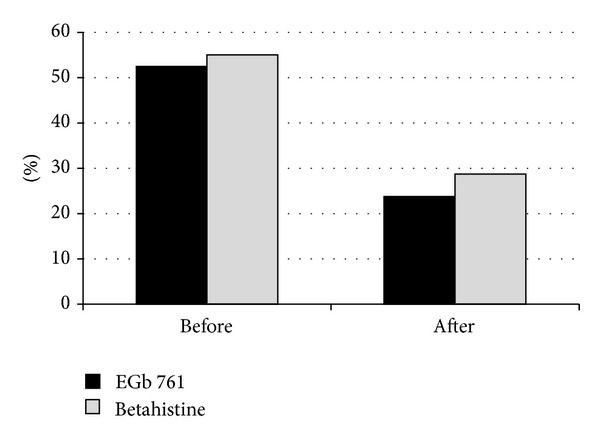
Proportion of patients with nystagmus before and after treatment.

**Figure 4:**
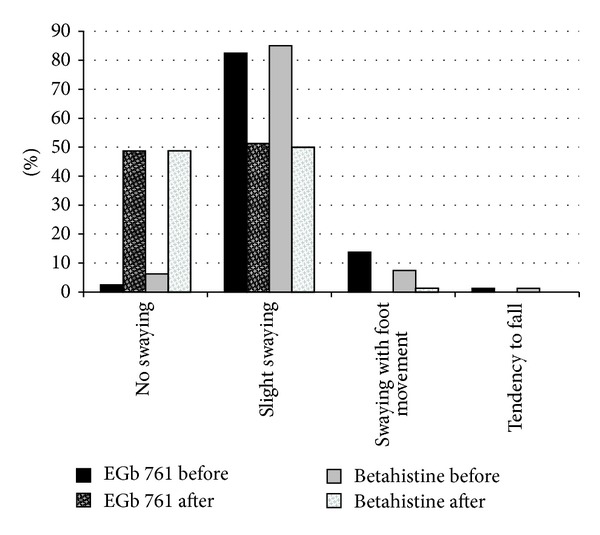
Proportion of patients with different grades of swaying in the Romberg test before and after treatment.

**Figure 5:**
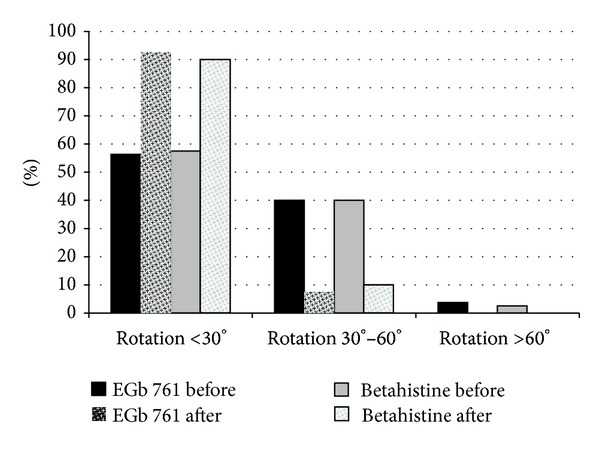
Proportion of patients with different grades of rotation in Unterberger's stepping test before and after treatment.

Patient-rated overall severity of vertigo (NAS) as well as symptoms of vertigo (VSS-SF) and disability due to vertigo (SDS) improved markedly in both treatment groups (Table 2, Figure 2). Similarly, clinician-rated global impression of change and neurootological findings indicated considerable improvements under both EGb 761 and betahistine treatments (Table 2, Figures 2, 3, 4, and 5). There were no significant differences between the two treatment groups with respect to treatment-related changes. The size of treatment effects did not vary with age, gender, clinical neurootological findings, or severity of symptoms.

### 3.2. Safety

During the treatment period and a subsequent two-day washout period, 27 adverse events (AEs) were reported for 19 patients in the EGb 761 group and 39 AEs were documented for 31 patients receiving betahistine. Blinded review could not rule out a causal relationship with the study medication for 6 AEs in 5 patients taking EGb 761 and for 18 AEs in 16 patients of the betahistine group. There was one serious AE in the betahistine group (spondylolisthesis) for which a causal relationship could be excluded. The most frequently observed types of AEs are listed in Table 3.

### 3.3. Discussion

In this randomized, double-blind, multicenter clinical trial, we found* Ginkgo biloba* extract EGb 761 and betahistine to be equally effective in the treatment of vertigo. We enrolled patients with unspecified vertigo, because, on one hand, specific vertiginous syndromes (e.g., Ménière's disease and benign paroxysmal positional vertigo) require specific treatments, and, on the other hand, both drugs are widely prescribed for vertigo not defined as part of a specific syndrome. In fact, betahistine is by far the most frequently prescribed drug for vertigo worldwide [6]. The results complement the findings from placebo-controlled trials of EGb 761 that demonstrated its clinical efficacy in vestibular and nonvestibular vertigo [15].

More than 70% of the patients were rated “much improved” or “very much improved” by their physicians after the 12-week treatment period. Interestingly, the patients' ratings of overall improvement (NAS) closely match the degree of change assessed by the comprehensive symptom scale (VSS-SF), with about 60% improvement over the initial scores. The patients' subjective ratings and the physicians' global impressions of change were strongly supported by the objective findings from clinical neurootological examinations: swaying in the Romberg test and rotation in Unterberger's stepping test were decreased considerably and spontaneous nystagmus was no longer found in half of the patients who had nystagmus before treatment. Constraints in daily life due to vertigo, which were rated as moderate at enrollment, were reduced and perceived as not more than mild after treatment.

With a sample size of 80 patients per treatment arm the study did not have statistical power to prove equivalence of the two treatments. The findings should therefore be interpreted as descriptive. Another limitation of our study is the lack of a placebo group as “negative” control. Taking into account that both treatments are evidence-based [7, 15] and high rates of marked spontaneous improvements are not very likely after an average duration of symptoms of approximately 2 years, there is reason to assume that the observed effects are mostly treatment-related and not mere placebo effects. There are numerically (not statistically significantly) more pronounced improvements in all outcome measures in the patients treated with EGb 761 compared to those receiving betahistine. As the likelihood of observing a difference between two treatment groups going in the same direction in 7 outcome measures just by chance when there is no real difference is less than 0.05, this could point to a slight superiority of EGb 761. However, subtle differences between the treatment groups in prognostic variables not documented at baseline and not completely balanced by randomization cannot be excluded, so this finding should be interpreted with caution.

The dosage of betahistine warrants some consideration, since, based on an open-label study, the use of higher daily doses has been suggested recently for the prevention of attacks of Ménière's disease [20]. This may be reasonable; a clear distinction must be made, however, between the prevention of attacks and the symptomatic treatment of existing vertigo. Systematic reviews found daily doses of 32 mg to 36 mg most effective in the symptomatic treatment of vertigo [7, 8]. While a review of trials in various vertiginous syndromes found similar effect sizes for 32 mg and 36 mg [7], the Cochrane review of betahistine in Ménière's disease found the strongest effects in high-quality placebo-controlled trials at 32 mg but no difference from placebo for 72 mg per day [8]. Similarly, another meta-analysis published recently found no advantage of 48 mg over 32 mg per day in parallel-group trials [21]. We therefore believe that we used an appropriate dose for the symptomatic treatment of vertiginous syndromes.

In the light of recent research, one mechanism likely to be involved in the antivertiginous action of EGb 761 deserves attention. Vertigo and the sensation of dizziness may result from labyrinth dysfunction or disconnection or impaired processing of information within the central nervous networks (vestibular, ocular, oculomotor, cortical, and cerebellar) involved in equilibrium and posture control [22]. Aging-associated loss of cortical neurons and integrity of fiber tracts as well as a slowing of information processing [23] may contribute to or enhance such dysfunction. Impaired intrinsic and synaptic mechanisms of neuronal plasticity [22, 24] are likely to prevent full compensation of disturbances in the vestibular system and to play a role in long-lasting vertiginous syndromes. EGb 761 has been shown to enhance neuronal plasticity by stimulating neurogenesis, neurite outgrowth, synaptogenesis, and synaptic function [25]. Of note, Lacour and colleagues [26] observed an accelerated recovery of synaptic density in the medial vestibular nuclei of EGb 761-treated cats after unilateral vestibular neurectomy.

Regarding safety and tolerability, EGb 761 seems to have some advantage over betahistine. No patients withdrew from treatment due to adverse events, but the total number of adverse events as well as the number of patients who experienced an adverse event was lower in the EGb 761 group than in the betahistine group.

## 4. Conclusion

This study provides evidence that* Ginkgo biloba* extract EGb 761 is at least as effective as the world's most frequently prescribed antivertiginous agent, betahistine, in the treatment of unspecified vertiginous syndromes.

## Figures and Tables

**Table 1:** Demographic data, rating scale scores, and clinical neurootological findings at enrollment; means ± standard deviations or numbers/percentages; *P* values (Wilcoxon test and Fisher's exact test*).

	EGb 761	Betahistine	*P* value
Gender			
Male	25/31%	22/27%	0.603*
Female	55/69%	58/73%	
Age [years]	57.5 ± 9.2	57.9 ± 8.4	0.561
Body mass index [kg/m^2^]	27.0 ± 4.2	28.3 ± 4.2	0.082
Duration of symptoms [months]	25.1 ± 37.3	22.5 ± 31.2	0.633
NAS	5.4 ± 1.4	5.2 ± 1.2	0.644
VSS-SF total score	24.2 ± 8.9	22.6 ± 8.5	0.239
VSS-V subscore	10.6 ± 5.5	9.9 ± 5.4	0.433
VSS-A subscore	13.5 ± 5.6	12.6 ± 4.9	0.447
SDS total score	15.2 ± 5.4	14.2 ± 5.4	0.263
Spontaneous nystagmus	42/52.5%	44/55%	0.874
Romberg test			
No swaying	2/2.5%	5/6.3%	0.447
Slight swaying	66/82.5%	68/85.0%	
Swaying/foot movement	11/13.8%	6/7.5%	
Tendency to fall	1/1.3%	1/1.3%	
Unterberger's stepping test			
Rotation <30°	45/56.3%	46/57.5%	1.000
Rotation 30°–60°	32/40.0%	32/40.0%	
Rotation >60°	3/3.8%	2/2.5%	

**Table 2:** Changes during the 12-week treatment period; means ± standard deviations; *P* values (Wilcoxon test).

	EGb 761	Betahistine	*P* value
NAS	−3.5 ± 1.8	−3.3 ± 1.7	0.704
VSS-SF total score	−14.7 ± 7.8	−13.4 ± 8.5	0.319
VSS-V subscore	−7.6 ± 5.0	−7.0 ± 5.2	0.432
VSS-A subscore	−7.1 ± 4.1	−6.4 ± 4.6	0.446
SDS total score	−9.4 ± 5.7	−8.3 ± 5.7	0.260
CGI change score	1.9 ± 0.9	2.1 ± 0.9	0.237

**Table 3:** The most frequently observed AEs in both treatment groups (at least three events in one treatment group).

System organ class—type of AE	EGb 761 (*n*)	Betahistine (*n*)
Gastrointestinal disorders:		
Dyspepsia and abdominal discomfort/pain	2	5
Nausea and vomiting	3	1
Diarrhea, frequent bowel movements infections, and infestations	1	3
Respiratory tract infections and nervous system disorders	5	9
Headache	3	5
